# Advanced sleep spindle identification with neural networks

**DOI:** 10.1038/s41598-022-11210-y

**Published:** 2022-05-10

**Authors:** Lars Kaulen, Justus T. C. Schwabedal, Jules Schneider, Philipp Ritter, Stephan Bialonski

**Affiliations:** 1 Department of Medical Engineering and Technomathematics, FH Aachen University of Applied Sciences, 52428 Jülich, Germany; 2 Independent researcher, Lessingstraße 65, 53113 Bonn, Germany; 3 Department of Psychiatry and Psychotherapy, University Hospital Carl Gustav Carus, Technische Universität Dresden, 01307 Dresden, Germany; 4 Institute for Data-Driven Technologies, FH Aachen University of Applied Sciences, 52428 Jülich, Germany

**Keywords:** Biomedical engineering, Computational models, Machine learning, Sleep

## Abstract

Sleep spindles are neurophysiological phenomena that appear to be linked to memory formation and other functions of the central nervous system, and that can be observed in electroencephalographic recordings (EEG) during sleep. Manually identified spindle annotations in EEG recordings suffer from substantial intra- and inter-rater variability, even if raters have been highly trained, which reduces the reliability of spindle measures as a research and diagnostic tool. The Massive Online Data Annotation (MODA) project has recently addressed this problem by forming a consensus from multiple such rating experts, thus providing a corpus of spindle annotations of enhanced quality. Based on this dataset, we present a U-Net-type deep neural network model to automatically detect sleep spindles. Our model’s performance exceeds that of the state-of-the-art detector and of most experts in the MODA dataset. We observed improved detection accuracy in subjects of all ages, including older individuals whose spindles are particularly challenging to detect reliably. Our results underline the potential of automated methods to do repetitive cumbersome tasks with super-human performance.

## Introduction

Sleep spindles are phenomena occurring in the brain of sleeping mammals^1^ that are associated with diverse neurological processes, such as cognitive abilities, including learning, memory consolidation and intelligence^1–5^. Changes in sleep spindle characteristics have been reported for various sleep disorders^6^, mental illnesses such as schizophrenia^7–9^, autism^10,11^ and bipolar disorder^12^, and neurodegenerative diseases such as Alzheimer’s disease^13,14^. These findings suggest that sleep spindles may serve as prognostic and diagnostic biomarkers for various diseases in the future^1^.

Spindles are brief, distinct bursts of rhythmic activity visible in electroencephalographic recordings (EEG) with frequencies in the range of 11–16 Hz (sigma-band) and a duration of at least 0.5 s, according to the American Academy of Sleep Medicine (AASM)^15^. Sleep spindles are generated in the thalamic reticular nucleus and relayed to the cortex by neuronal feedback loops^1^. The gold standard for spindle detection is spindle identification by trained experts who visually inspect and annotate EEG signals. This manual process is known to be time-consuming and therefore expensive, and is thus a limiting factor for large-scale spindle studies. Moreover, inconsistencies and disagreements among experts introduce noise in spindle annotations (label noise). Indeed, only substantial agreement was reported when an expert inspected the same data multiple times (*intra-rater reliability*, Cohen’s $\kappa =0.66$), and the agreement between multiple experts (*inter-rater reliability*) was only moderate ($\kappa = 0.52$)^16^.

An automated system for the detection of spindles that mimics an expert scorer would determine spindles consistently, but it would be limited by the level of disagreement among experts. This challenge can be addressed by having a group of experts separately identify sleep spindles in order to form a group consensus (*expert consensus*)^16^. By increasing the number of experts, such a consensus converges to generalizable annotations^17^. For sleep spindle identification, it was estimated that 2–3 experts were required to achieve a consensus of ‘substantial’ reliability, and at least 4 or more experts were needed to reach ‘almost perfect’ reliability^16^. Publicly available datasets of spindle annotations (such as MASS-SS2 with 19 participants about 23.6 years old, see Montreal Archive of Sleep Studies^18^) contain recordings of several subjects that are annotated by only one or two expert scorers. Notably, the DREAMS dataset^19^ was scored by five expert scorers but only comes from eight participants that were about 45.9 years of age^20^, thus limiting the generalization of systems trained thereon. Recently, in a remarkable crowdsourcing effort, the MODA (Massive Online Data Annotation) dataset was created in which EEG recordings from 180 subjects were scored for spindles by a median number of 5 experts (more than 95% of the data was annotated by at least 3 experts)^21^. Among the investigated methods for automated spindle identification, the algorithm A7^22^ achieved the highest level agreement with the expert consensus with a performance similar to an average expert scorer. However, as the consensus reduces the label noise substantially, we entertain the hypothesis of an automated system that exceeds individual expert scorers and replaces the manual annotation process, thereby enabling large-scale spindle studies.

The algorithms presented in the MODA study, including A7, are based on engineered features which make use of well-known physiological and physical properties of sleep spindles as they appear in the EEG, such as spindle amplitudes and frequencies^7,8,22–26^. The past years have shown that such feature-based methods are inferior to methods of deep learning with neural networks, especially for problems of object or event detection^27–29^. Recent studies have confirmed this trend by training a variety of neural network architectures on minimally processed EEG signals: neural-net-based approaches report superior performance with respect to the F1 score, a quantity usually evaluated at a 20% overlap between algorithmically-detected and expert-annotated spindle. *DOSED*^30^ achieved an F1 of about 0.75, 0.50, and 0.45 when evaluated on a young, middle-aged, and older cohort. Exceeding these results, *SpindleNet*^20^ achieved an F1 of 0.82 when trained on a young cohort, but reached only 0.48 on DREAMS, whose participants also have a variety of sleep pathologies. *RED-CWT* and *RED-Time*^31^, and *SpindleU-Net*^32^ achieved comparable performance of about 0.83 on the MASS-SS2 dataset; SpindleU-Net was also evaluated on DREAMS and exceeded previous results by reaching an F1 of 0.74.

In this contribution, we introduce a deep neural network model called *SUMO*^33^ (*S*lim *U*-Net trained on *MO*DA) that approximates the expert consensus derived from the MODA dataset to identify sleep spindles. The network architecture is inspired by U-Net^34^, a deep neural network that has been used with great success in the field of image segmentation^35^. The model complexity of SUMO is low compared to aforementioned deep learning models, which allows for data-efficient training on the MODA dataset. We demonstrate that our model surpasses the accurateness of the A7 algorithm and the average expert to identify spindles compared to the group consensus. Downstream measures (spindle duration and density) derived from the model’s predictions correlated stronger with the expert consensus compared to A7. We consider approaches such as ours to be promising for the future of automated sleep spindle identification.

## Materials and methods

### EEG and spindle dataset

We obtained two datasets to train and study the model. The first dataset contained EEG recordings from the Montreal Archive of Sleep Studies (MASS)^18^, which are publicly available^36^. Retrieval and analysis was approved by the local ethics committee at Technische Universität Dresden, Germany (permit number BO-EK-50012021). All analytical methods were carried out according to the ethical principles of the Declaration of Helsinki^37^ and the recommendations on good clinical practice^38^. The second dataset consisted of freely available crowdsourced spindle annotations from the Massive Online Data Annotation (MODA) study^21^. Of the 200 individual recordings contained in MASS, the MODA study selected 180 recordings of C3-LE or C3-M2 EEG channels. Recordings were split into a younger (24 years mean age) and an older (62 years mean age) cohort to make annotations. Each recording was divided into several 115 s long segments containing N2 sleep without artifacts. Ten segments were randomly sampled from each recording in a subset of 30 recordings (from 15 younger and 15 older subjects), and three segments were randomly sampled from each of the remaining 150 recordings. After band-pass filtering and downsampling, these segments were exhaustively scored by 47 certified EEG technicians. A median of five EEG technicians reviewed any one segment. Available to use was an expert consensus of spindle annotations formed from the annotations of the EEG technicians. The method by which the expert consensus was formed as well as other details can be found in Lacourse et al. (2020)^21^.

#### Signal preprocessing

We downsampled each EEG segment to 100 Hz using a phase-preserving, 10-th order Butterworth filter (passband 0.3–30 Hz) according to the MODA study. In addition, we z-transformed each segment (zero mean, unit variance) before further processing to decrease inter-individual differences in the recording modalities.

### Training and test sets

We divided the dataset into a training and a test set (see Table 1) guided by the following principles. All segments of any one individual shall belong to either the training or the test set. The ten-segment individuals shall belong to the training set. The test set shall have equal numbers of younger and older individuals. Algorithm A7 shall show an average performance on the test set segments.

The last principle was added because published results of A7 were obtained on the whole dataset, for which we were unable to generate an out-of-sample prediction of our model. To split off a test set that did not bias A7, we randomly drew 25 test set candidates from the distribution defined by the rest of the above principles. We evaluated A7 on each candidate and determined its F1 score (see “Evaluation of spindle detections.” section). From all candidate test sets, we chose the one on which A7 reached the median F1 score of the sample distribution.

**Table 1:** Training- and test-splits of individuals and EEG segments, respectively. We divided all individuals in the MODA dataset into a training and test set such that, for the test set, individuals and segments were divided equally between age groups. Among a set of random splits satisfying this equality, we selected the split with median performance for algorithm A7.

	cohort	training	test		sum
			younger	older	
individuals	younger	82	18	0	100
	older	62	0	18	80
	sum	144	18	18	180
segments	younger	351	54	0	405
	older	291	0	54	345
	sum	642	54	54	750

### Model architecture

**Figure 1:**
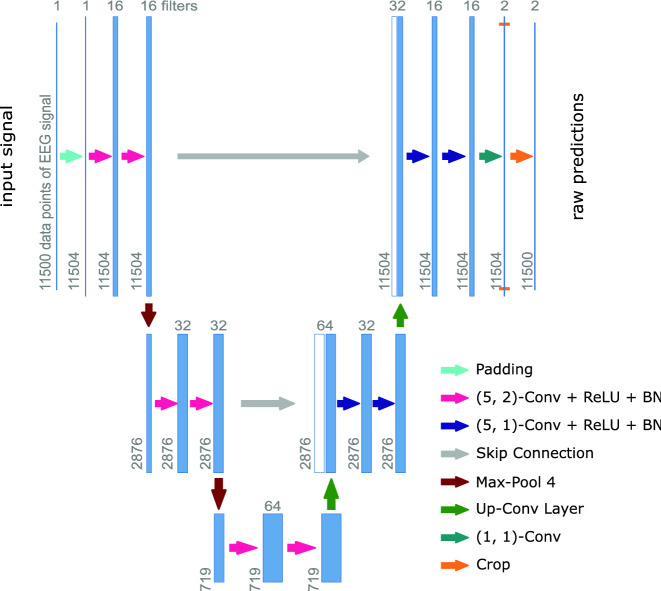
Architecture of the SUMO model. Each box represents a feature map with the number of filters given on top. The operations conducted are represented by the colored arrows. Convolutions are described using the format *(k, d)-Conv* with *k* denoting the kernel size and *d* the dilation size. ReLU: rectified linear unit; BN: batch normalization. Up-Conv Layer: nearest-neighbor upsampling with factor 4 followed by a (4, 1)-Conv.

The architecture of the model *SUMO* was inspired by U-Net^34^, which is a fully-convolutional network. SUMO consisted of a branching encoder making up the left side of the U followed by a converging decoder making up the right side (see Fig. 1). The encoder compressed temporal features in a single EEG segment into an increasing number of context channels through several levels of compression. The decoder expanded the context into temporal information while reducing the number of context channels in a symmetric number of expansion levels. After each compression, a branch was fed into the matching expansive level through shortcut connections (cf. Fig. 1). The output of the last layer represented raw per data point probabilities of being part of a spindle or not (no-spindle). These probabilities were either fed into the loss function or underwent further processing to predict spindle annotations.

Each level of compression and expansion consisted of two composite layers of convolution^39^, rectified linear unit^40^, and batch normalization^41^. The two convolutions had the same number of channels. The compression step on the left side of the U was implemented by max-pooling the composite-layer output. The expansion step (right side) was achieved by nearest-neighbor upsampling followed by a convolution mapping to half the number of input filters. This half was then supplemented by concatenating the output activations from the matching contracting step in the left side of the U.

When predicting spindle annotations, the raw output probabilities in the spindle and no-spindle channel were temporally smoothed with a moving-average filter and converted to spindle indicator values by taking the point-wise maximum. Consecutive positive indications were then joined to form spindle annotations constituted by a starting sample and a duration. The width of the moving-average filter was a hyperparameter that we optimized (see “Results” section).

### Training

Before training, we divided all training segments into training and validation sets using a six-fold cross validation procedure for hyperparameter optimization and early stopping. Similar to the test-training splits, the six folds were split on a by-individual basis wherein the 10-segment individuals were equally divided across the folds. This means that the 144 subjects of the training set (see Table 1) form six folds of 24 subjects each, where a model is trained on five folds (120 subjects) and evaluated on the remaining fold (24 subjects). Six separate training sessions were carried out to perform the six-fold cross validation. For each session, we initialized the trainable network parameters using Kaiming’s procedure of uniform initialization^42^. Feeding the network with minibatches of training examples (12 segments), we evaluated the output and the expert-consensus annotations using a generalized dice loss function^43^. The resulting error signals were backpropagated and parameters were updated using Adam stochastic gradient descent with parameters $\beta _1=0.9$, $\beta _2=0.999$, $\epsilon =10^{-8}$, and learning rate $\alpha =0.005$^44^. After processing each sample in the training data once (i.e., after one training epoch), we computed the network’s F1 score (see next section) for the validation set which was one of the six folds. If the F1 score did not improve over the course of 300 training epochs, or if the 800-th training epoch was reached, the training was stopped. We then stored the parameters of the best performing models (one model for each validation fold) from the training session for further processing.

### Evaluation of spindle detections

We evaluated the performance of our model in an *analysis by event* and an *analysis by subject*. The analysis by event reflects the primary objective of the model to reliably identify individual sleep spindles. Following Warby et al.^45^, we compared a detected set of spindles to the expert consensus (EC) on a by-spindle (by-event) basis. For each EC spindle, we determined the temporal overlap of the closest detected spindle relative to the joined duration of both. If that relative overlap exceeded a threshold, we counted a true positive (TP). Else, we counted a false negative (FN). Each detection that did not sufficiently overlap with an EC spindle was counted as a false positive (FP). We note that an alternative to this by-event evaluation would be to evaluate each data point in a spindle separately. However, such an approach would put additional weight on long spindles because of temporal correlations, which we wanted to avoid.

From the evaluations, we computed the recall, $\text {TP}/(\text {TP}+\text {FN})$, the precision, $\text {TP}/(\text {TP}+\text {FP})$, and the F1 score, $2\cdot \text {TP}/(2\cdot \text {TP}+\text {FP}+\text {FN})$, to quantify the quality of spindle detection. All these metrics depend on the chosen overlap threshold that determines TP, FN, and FP. Thus, as a parameter-free metric, we also determined $\overline{\text {F1}}$ as the integral of F1 scores over overlap thresholds. Finally, for a more detailed *by-event* analysis, we studied SUMO’s performance stratified by age cohorts.

In our analysis by subject, we investigated the usefulness of the predictions for downstream measures such as average spindle characteristics which are of interest to sleep researchers. From the predicted spindles, we determined average spindle duration and spindle density per subject. We studied how much these characteristics were correlated with those derived from the expert consensus.

## Results

### Hyperparameter optimization

**Figure 2:**
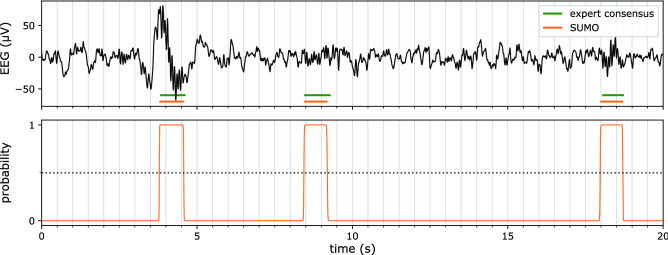
Comparison of SUMO predictions with expert consensus on an exemplary EEG segment. Top panel: A 20 s long segment of N2-sleep EEG from the test set shows three spindles marked by the expert consensus (green marks). Our trained SUMO model detects all three spindles with about 90% overlap (orange marks). Bottom panel: The predictions are generated by thresholding SUMO’s output probabilities (orange line) with a threshold of 0.5 (black line).

We explored how accurate SUMO could predict the expert consensus depending on the architecture of the model, i.e. the number of levels, the maximal receptive field, the number of channels, and the width of the moving-average filter applied to the net’s raw output. We focussed on these parameters hypothesizing their importance to accurately predict spindles, which show characteristic time scales in their occurrence, duration, and rhythm. For a given configuration, we carried out a six-fold cross validation by training six SUMO models that were independently initialized. After one training epoch of 45 training steps (equivalent to the size of the training set), we applied the model in its current state to the validation set to determine the F1 score dependent on the overlap threshold. From this dependence, we computed $\overline{\text {F1}}$ as a parameter-free metric of the model performance. If exceeding previous $\overline{\text {F1}}$ values, we noted the new value and stored the parameters as the new candidate for the best model. After about 300 training epochs, the F1 score did typically not improve further and the training stopped. The training was also stopped after 800 training epochs which occurred in about 50 individual training sessions. In Fig. 2, we show an exemplary spindle prediction of a trained SUMO model applied to a 20 s long EEG segment together with the expert-consensus spindles. In the example, all three spindles were detected with an overlap of about $90\%$.

After all six training sessions were concluded, we compared the average across the best $$\overline{\text {F1}}$$ from each fold to $$\overline{\text {F1}}$$ averages obtained for other network configurations. We varied the number of levels of the architecture, and within each level the width of the max-pooling operation to fine tune their receptive fields. We also changed the model capacity by varying the number of convolutional filters per level. We varied the number of levels of the model between 2 ($$\overline{\text {F1}}$$$$=0.599\pm 0.017$$) and 5 ($$\overline{\text {F1}}$$$$=0.635\pm 0.015$$), and found a shallow optimum of $$\overline{\text {F1}}$$$$=0.638\pm 0.021$$ for an architecture consisting of 3 levels. Here, max pooling operations went across 4, 4, 2, and 2 data points in consecutive levels of the model. For 3-level models, we varied the width $$w_2$$ of the second max pooling while keeping the first max-pooling width $$w_1$$ at 4. Between $$w_2=1$$ (receptive field of 1.48 s), where we found $$\overline{\text {F1}}$$$$=0.611\pm 0.027$$, and $$w_2=8$$ (6.24 s, $$\overline{\text {F1}}$$$$=0.633\pm 0.027$$), we found a shallow optimum of $$\overline{\text {F1}}$$$$=0.644\pm 0.015$$ at $$w_2=4$$ (i.e. 3.52 s). When doubling the number of convolutional filters in the 5-level model (number of filters per level: 32, 64, 128, 256, 512), we observed $$\overline{\text {F1}}$$$$=0.629\pm 0.022$$ compared to $$\overline{\text {F1}}$$$$=0.635\pm 0.015$$ without doubling (filters per level: 16, 32, 64, 128, 256). We did not observe large variations in $$\overline{\text {F1}}$$ when further increasing the number of convolutional filters and thus used the number of filters as depicted in Fig. 1. Finally, we optimized the width of the moving-average filter searching in a range between 10 and 200 data points (0.1-2 s). We found a shallow optimum at 42 data points where we reached $$\overline{\text {F1}}$$$$=0.646$$.

In total, we trained about 100 such configurations and found their F1 scores to be remarkably stable across folds. We thus determined the optimal model configuration within our search space to consist of three levels, each one pooling across 4 time points. The raw output was further processed with a 0.42-s wide moving-average filter before thresholding in order to determine spindle predictions.

### Analysis by event

We then went on to compare the performance of the SUMO model to algorithm A7 and the expert consensus (see results in Table 2). First, we extracted recall, precision, and F1 score (for 20% overlap threshold) of the average expert and A7 from Lacourse et al.^21^, supplementary Table 2. We also computed these metrics for A7 on the hold-out test set. Comparing A7 performance on the test and the whole dataset, we found that all test set metrics were equal to, or numerically exceeded the metrics obtained on the whole dataset.

We also evaluated SUMO in its best-performing configuration on the test set and found that its F1 score exceeded A7 by 10 base points on the younger cohort, and by 8 base points on the older cohort. By comparing all SUMO metrics from the test set to those on the validation and training set (evaluated for the respective fold), we found no signs of overfitting across all three sets.

**Figure 3:**
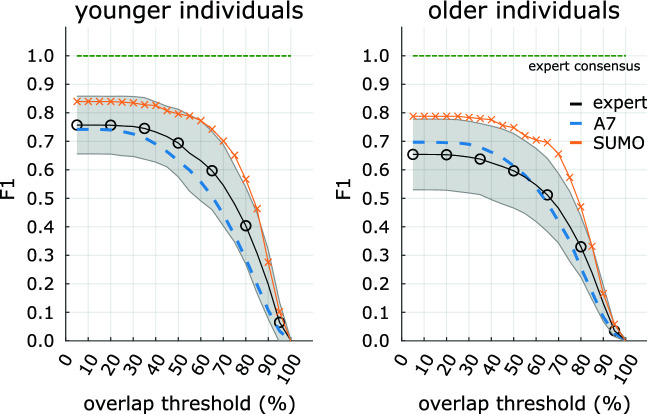
Overlap threshold dependency of F1 scores computed against the expert consensus. The F1 scores of algorithmic (A7: blue dashed line, SUMO: orange crosses) and expert (mean: black circles, standard deviation: gray area) detections decrease monotonically with the overlap threshold for younger (left panel) and older (right panel) individuals. At all thresholds, SUMO shows a higher F1 score compared to A7 as well as most individual experts. SUMO F1 scores were computed on the test set, which we designed to mimic the whole dataset with respect to the A7 performance. Expert and A7 F1 scores were reproduced from Figure 2 in Lacourse et al.^21^, licensed under CC BY 4.0 International^46^. As explained therein, the F1 scores of A7 were computed across the whole dataset, whereas F1 scores for individual experts were computed across the scored subset, and against all remaining experts. For comparison, the green dashed line indicates the expert consensus which serves as the ground truth.

We analyzed how our best model compared with average-expert and A7 detections for overlap thresholds different from 20%. We extracted the comparative performance values from Figure 2 in Lacourse et al. (2020)^21^ and overlaid the F1 scores from our best model on the test set. As shown in Fig. 3, we found that our F1 scores were higher compared to the average expert and A7 for all overlap thresholds. In the case of the older individuals, our F1 score was consistently one standard deviation better (i.e., above the gray area in Fig. 3) than the average expert except for overlap thresholds larger than 90%.

**Table 2:** Recall, precision, and F1 score of mean expert, A7, and SUMO in younger and older individuals. Our best SUMO model numerically exceeds the mean-expert and A7 scores in all categories. The test set performance of A7 exceeds its values on the full dataset except for the precision metric. All metrics were computed with a 20% overlap threshold. Numbers in parentheses denote standard deviations across the folds of the 6-fold cross validation. The individual experts were evaluated against the leave-one-out expert consensus.

detector	data	recall			precision			F1 score		
		both	younger	older	both	younger	older	both	younger	older
mean expert	all	0.72	0.76	0.66	0.78	0.81	0.74	0.72	0.76	0.65
A7^22^	all	0.73	0.75	0.70	0.71	0.73	0.69	0.72	0.74	0.70
A7^22^	test	0.76	0.78	**0.73**	0.70	0.70	0.69	0.73	0.74	0.71
SUMO	test	**0.79**	**0.82**	**0.73**	**0.85**	**0.85**	**0.85**	**0.82**	**0.84**	**0.79**
SUMO	val	0.81 (0.01)	0.85 (0.02)	0.75 (0.01)	0.83 (0.02)	0.83 (0.03)	0.83 (0.01)	0.82 (0.01)	0.84 (0.02)	0.79 (0.01)
SUMO	train	0.82 (0.02)	0.85 (0.02)	0.76 (0.03)	0.83 (0.02)	0.83 (0.02)	0.83 (0.02)	0.82 (0.00)	0.84 (0.01)	0.79 (0.01)

Maximum values obtained on the test set are shown in bold.

### Analysis by subject

Many sleep studies identify sleep spindles to derive characteristics such as the number of spindles per minute (spindle density), or the average spindle duration. We tested how these characteristics derived from automatic spindle annotations compared to the expert consensus.

**Figure 4:**
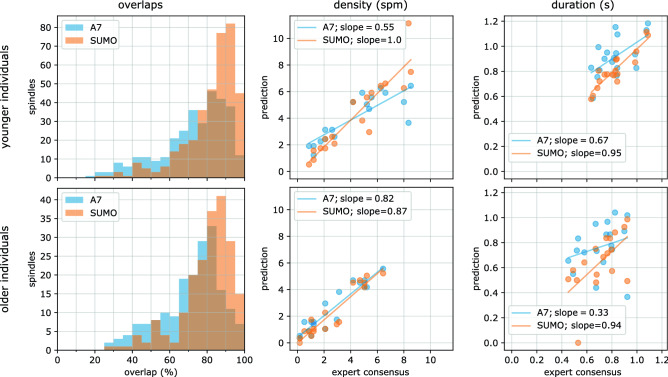
Overlap distribution, and spindle density and duration compared with expert consensus. Left panels: The frequency distribution of spindle predictions at specific overlaps computed for all spindles in the test set is more tightly peaked for SUMO (orange) compared with A7 (blue). The peak of the SUMO model is also shifted towards higher overlap percentages. Middle panels: Correlation analysis between per-individual spindle density from the expert consensus and predictions (SUMO, A7) indicate a flattened slope in younger individuals for A7 clearly deviating from 1. Right panels: Similarly comparing spindle durations, A7 also shows a flattened slope.

We determined spindle density and duration for each individual in the test set from the expert consensus, A7, and the SUMO model presented in the previous section. We then analyzed their linear dependency by fitting a linear model to the relationship of algorithm-derived and consensus characteristics and noted the slope *m*. This slope should be 1 to accurately indicate changes of the given spindle characteristic caused by an intervention, for example. As a measure of precision of the predicted spindle characteristic, we determined the level of correlation using Pearson’s r. Resultant r-values were compared across the two algorithms after applying Fisher’s z-transform^47^. The results of our analyses are shown in Fig. 4 (middle and right column) and Table 3.

#### Spindle density

The expert consensus showed a bimodal distribution of spindle densities with modes at about 1 and 5.25 spindles per minute. Both algorithm-derived spindle densities showed a high level of correlation with the expert consensus, with A7 showing $r^2=0.68$ for younger and $r^2=0.88$ for older participants, and SUMO showing $r^2=0.84$ and $r^2=0.89$. When comparing the z-transformed r-values, we found no statistically significant difference at $\alpha =0.05$.

The slope of A7-derived spindle densities deviated numerically more from 1 than SUMO-derived densities, especially for younger individuals with $m_{A7}=0.55$.

#### Spindle duration

In the analysis of spindle duration, we found much weaker correlations compared to the density results presented above. A7 showed $r^2=0.35$ for younger and $r^2=0.07$ for older participants, and SUMO showed $r^2=0.82$ and $r^2=0.37$. A statistical comparison (based on Fisher’s z-transform) gave some evidence that SUMO derived durations correlated stronger than A7 for younger individuals ($p=0.02$), but not for older ones ($p=0.23$).

When comparing the slope, we found that the A7 fit deviated markedly from an optimal slope showing $m=0.67$ for younger and $m=0.33$ for older individuals. In both cases, the SUMO spindle durations showed a slope of about $m=0.95$.

**Table 3:** Correlation coefficient $r^2$ between spindle properties derived by automatic methods (A7 or SUMO) and the consensus of the expert group.

Method	Density		Duration	
	Younger	Older	Younger	Older
A7^22^	0.68	0.88	0.35	0.07
SUMO	0.84	0.89	0.82	0.37

Spindle density (spindles per minute) and average spindle duration were determined for each subject of the test set based on spindles identified by A7, SUMO, and the expert consensus. For each age cohort (younger, older), correlation coefficients between spindle properties based on the expert consensus and the automatic methods were determined.

## Discussion

We trained the U-Net-type neural network SUMO on the MODA dataset to reliably identify sleep spindles in the electroencephalographic recordings in a test set stratified into younger (24 years mean age) and older (62 years mean age) individuals. Our hyperparameter tuning optimized $\overline{\text {F1}}$, the integral of F1 scores over overlap thresholds, computed from the model detections and the expert-consensus spindles. We compared the performance metrics $\overline{\text {F1}}$, as well as precision, recall, and F1 scores for a range of overlap thresholds of our trained model with the A7 algorithm as well as individual experts reported in Lacourse et al.^21^. Note that we did not evaluate the individual expert performance on the test set. Furthermore, we compared by-subject estimates of clinically-important spindle metrics (spindle density and average duration) derived by SUMO and A7 to those determined from the expert-consensus spindles using correlation analysis.

Spindles detected by our model showed, on average, larger overlap with those of the expert consensus than A7 (see Fig. 4, left column). Better agreement with expert-consensus spindles was also visible in F1 scores which started to decrease at larger overlap thresholds for SUMO compared to A7 (see Fig. 3). In line with previous studies^21,22,31,45^, we evaluated the performance of our model for an overlap threshold of $20\%$. SUMO achieved an F1 score of 0.82 for the subjects in out-of-sample data (test set, both cohorts, see table 2, third-last column). This surpassed the A7 algorithm (0.73) on the test set and the average expert (0.72) on all data. Our model’s performance is comparable to a limited extent with those reported for other deep learning models at 20% overlap which were, however, trained on different datasets (with different age distributions) and predominantly based on annotations of individual experts. Reported F1 scores for models evaluated on datasets of young individuals were comparable to SUMO’s F1 score of 0.84 for the young cohort (F1 scores averaged over individual experts: 0.83 (RED-CWT and RED-Time)^31^, 0.83 (SpindleU-Net)^32^, 0.83 (SpindleNet)^31^, 0.80 (DOSED)^31^). These observations may suggest that the accurateness of automated spindle detection has approached a limit imposed by non-negligible inter- and intra-rater reliability of experts who annotated the data. They also provide evidence of the superiority of deep learning methods compared to classical feature-based approaches such as A7, which achieved an F1 score of 0.74 for the younger cohort (see table 2). When evaluated on datasets of older individuals, reported performance of other deep learning models was lower than SUMO’s F1 score of 0.79 for the older cohort (0.74 (SpindleU-Net)^32^, $\text {F1}<0.60$ (DOSED)^30^). We note, however, that datasets of older individuals often include patients with various sleep pathologies, introducing additional factors that may limit comparative assessment of results. Nevertheless, the trend towards decreased detection performance was also visible for A7 (F1 score of 0.71, see table 2). The difference in detection performance between age groups may be explained by spindle amplitude and duration. Both are known to decrease with age^48,49^, which can make it more difficult for human scorers and automated methods to delineate spindles from the surrounding background signals in older individuals^16^. From a clinical perspective, however, we consider the reliable detection of sleep spindles in older subjects to be of particular importance as sleep disorders become more frequent with age. The availability of large datasets with expert-consensus annotations may prove particularly valuable for the development of spindle detectors and may explain SUMO’s superior performance for this age group.

Detecting individual spindles is only of indirect importance in the evaluation of a clinical study, for example: to evaluate clinical outcomes, statistical characteristics of spindles are compared across subjects, raising the question of how imperfect detection algorithms reflect on such statistical estimates. In our by-subject analysis of the density and mean duration of spindles, we evaluated the correlation as well as the slope (from a linear model) between consensus and automated estimates by A7 and SUMO within age groups. Spindle densities computed from the expert consensus showed high levels of correlation with those estimated from A7 ($r^2 \ge 0.68$) and SUMO ($r^2 \ge 0.84$, see table 3). Spindle durations, on the other hand, correlated strongly for SUMO ($r^2 = 0.82$) in the younger cohort, but only moderately for A7 ($r^2=0.35$), whereas for the older cohort both methods yielded low correlations (0.37 and 0.07, respectively). Slopes of the linear models were close to one for SUMO ($m\ge 0.94$) for all spindle characteristics and age cohorts except for the spindle density of the younger cohort ($m=0.87$). All slopes for A7 were lower than for SUMO and achieved the largest value for the spindle density of the older cohort ($m=0.82$) and the lowest for the spindle duration of the older cohort ($m=0.33$). The results indicate that the spindle density can be estimated with higher accuracy compared to the mean spindle duration, and that A7 yields a biased estimator of these quantities. More complex spindle statistics may show additional systematic errors, and we recommend that the accurate automatic estimation of specific metrics should be verified before relying on their estimates. We speculate that spindle duration may sensitively depend on large overlap thresholds between predicted and actual spindles in order for predicted and actual durations to become similar. As the overlap threshold increased in both age cohorts, the F1 score decreased for A7 and SUMO, albeit more rapidly for A7 than for SUMO and to lower values for the older cohort than the younger cohort (see Fig. 3). This may explain the low levels of correlation of A7 or SUMO derived spindle duration for the older cohort, calling for methods that can more accurately identify sleep spindles at large overlap thresholds. Thus, we recommend future studies to evaluate spindle detectors at larger overlap thresholds (e.g., at 40%).

Our model has low model complexity and few hyperparameters since all layers are fully convolutional. Small model complexity allowed the model to be trained in a data-efficient way which prevents overfitting on small to medium-sized datasets such as MODA and eliminates the need for sophisticated regularization strategies (e.g., dropout). Indeed, we did not find evidence of overfitting since the F1 score obtained on the training set did not exceed the ones obtained on the validation and test sets (0.82, see third-last column in table 2). Various architectural changes (e.g., number of convolutional filters, the number of levels of the U, or the receptive fields of neurons of the last levels) did not sensitively affect the accurateness of spindle detection. While increasing the number of U-Net levels above three did not change F1 scores, increasing the receptive field of neurons (i.e. the length of an EEG segment a neuron has access to) of the third level above 3.5 s led to a slight decrease of F1 scores. This may indicate that particular time scales are relevant for the model to yield reliable spindle predictions. As spindle events usually show a duration between 0.5 and 1 s^21^, time scales larger than 3–4 s may not be informative for the model to reliably delineate spindle events from surrounding EEG activity. Thus, deeper levels of the U that integrate larger time scales may be ignored by the model.

The present study is limited in several respects. As previous methods for automated spindle detection, our model was trained to detect spindles in univariate time series (channel C3). Such a univariate approach does not localize sleep spindles (scalp localization^50^), a task desirable when studying the topographical distribution of spindles. While in principle our model can be used repeatedly to identify spindles in other channels, it remains to be investigated how accurate such detections are. Accurate localization of spindles could be achieved by multivariate models that would base their detections on all EEG channels. To develop and assess such models, we consider the creation of datasets with expert-consensus annotations for all EEG channels as the most important prerequisite. Another limitation shared with previous work^22,31^ is also related to the data used to train our model. Data were selected from N2 sleep stages of healthy individuals and were free from EEG artifacts. Ordinary EEG recordings will not meet these criteria, and spindles are also studied in N3 sleep. Models that accurately identify sleep spindles in N2 and N3 sleep and account for artifacts could be developed as datasets with corresponding expert-consensus annotations become available. Another limitation is related to the interpretation of expert-consensus spindles as objective truth. It is not clear whether spindles detected by models but not identified by the expert consensus nevertheless are mechanistically identical or may even reflect other biologically meaningful processes^21^. Furthermore, if an individual expert identified “true” spindles much more accurately than other experts, the expert consensus would be of lesser quality compared to this best individual expert. Since our model approximates an expert consensus, detected spindles are only objective in the sense that many experts would agree on them. Finally, we consider comparisons with previously published deep learning models on the MODA dataset as informative for future model development. To reduce difficulties for such comparative assessments in future studies, we publish our model together with detailed instructions^33^.

Our results underline the potential of deep learning models to automate the cumbersome task of spindle detection with super-human performance. We consider the availability of large datasets with expert-consensus annotations as key driver for the progress of methods towards objective and reliable spindle detection. We are confident that automatic spindle detection will continue to improve, saving staff and sleep researchers time and facilitating large-scale sleep studies.

## Data Availability

The MODA dataset analyzed in the current study is described in Lacourse et al.^21^ and is publicly available on the Open Science Framework (OSF)^51^. The MASS dataset^18^, which contains the EEG recordings, is publicly available and can be obtained from the Montreal Archive of Sleep Studies web page^36^. The source code of the SUMO model is available on GitHub^33^.
